# Antiepileptic and Neuroprotective Effects of Oleamide in Rat Striatum on Kainate-Induced Behavioral Seizure and Excitotoxic Damage via Calpain Inhibition

**DOI:** 10.3389/fphar.2017.00817

**Published:** 2017-11-21

**Authors:** Hye Yeon Nam, Eun Jung Na, Eunyoung Lee, Youngjoo Kwon, Hwa-Jung Kim

**Affiliations:** College of Pharmacy, Graduate School of Pharmaceutical Sciences, Ewha Womans University, Seoul, South Korea

**Keywords:** oleamide, kainic acid, epilepsy, calpain, neuroprotective effect

## Abstract

Oleamide was first known as a sleep-inducing fatty acid amide, and later shown to have wide range of neuropharmacological effects upon different neurochemical systems. However, the effects of oleamide on brain damage have scarcely been studied, and the molecular mechanisms and sites of its action remain elusive. Kainic acid (KA) has been used to produce an epileptic animal model that mimics human temporal lobe epilepsy and to induce calpain-activated excitotoxicity, which occurs in numerous neurodegenerative disorders. In this study, we examined whether oleamide protects against the KA-induced excitotoxic brain damage accompanied by behavioral seizure activity and neuronal cell death. Moreover, whether these effects of oleamide were mediated by calpain activity-related cellular mechanisms was investigated. KA-induced epileptic rats were produced by an intrastriatal injection of KA (5 nmole). Oral administration of oleamide (0.5, 2, and 10 mg/kg) 30 min prior to the KA injection showed dose-dependent inhibition of the KA-induced behavioral seizure activities that were monitored starting from 60 to 180 min post-surgery. Further repetitive oral administration of oleamide (once per day) for the next 4 consecutive days post-KA injection produced significant neuroprotection against the disrupted neuronal integrity that resulted from KA-induced excitotoxic damage that was also demonstrated by staining of striatal tissue sections with cresyl violet, hematoxylin/eosin, and fluoro-Jade B. In addition, oleamide blocked the KA-induced cleavage of cyclin-dependent kinase-5 coactivator (Cdk5-p35) and collapsin response mediator protein-2, which are believed to be mediated by calpain activation in striatal tissues dissected from KA-induced epileptic rats. Oleamide also reversed the KA-induced reduction in expression of an endogenous calpain inhibitory protein, calpastatin, and a marker of synaptic activity, synapsin-II. The hypothesis that oleamide could induce direct calpain inhibition was further investigated using *in vitro* calpain assays in both brain tissue and a cell-free and calpain-overexpressed neuronal cell system. These findings together suggest that oleamide has protective effects against excitotoxicity-induced neuronal death and behavioral seizure, partly via its direct calpain inhibitory activity.

## Introduction

Oleamide (*Cis*-9,10-octadecenoamide) is a centrally acting fatty acid amide that belongs to the family of endogenous lipid signaling molecules that includes endocannabinoids, anandamide, N-palmitoylethanolamine, and N-oleoylethanolamine (Fowler, 2004). Oleamide was first found to exist in the cerebrospinal fluid of sleep-deprived animals and act as an endogenous sleep-inducing substance (Cravatt et al., 1995). Besides inducing sleep, systemic administration of exogenous oleamide has been shown to produce a variety of central nervous system (CNS) effects (Boger et al., 2000b; Leggett et al., 2004), including elicitation of hypothermia (Fedorova et al., 2001), analgesia, memory (Murillo-Rodriguez et al., 2001; Akanmu et al., 2007), food intake (Martinez-Gonzalez et al., 2004), hypo-locomotion (Huitron-Resendiz et al., 2001), and reduction of pentylenetetrazole-induced epileptic behavior (Wu et al., 2003; Solomonia et al., 2008). Furthermore, it was recently reported that oleamide reduces amyloid-β (Aβ) accumulation via enhanced microglial phagocytosis and suppresses inflammation after amyloid Aβ deposition (Ano et al., 2015).

Although a wide range of neuropharmacological actions of oleamide have been suggested in several neurotransmitter systems, its effects on brain damage are less well studied and its mechanisms of action remain elusive. The endocannabinoid system is known to play a role in the cell death/survival decision and to improve glutamate homeostasis, thus reducing excitotoxicity (Zoppi et al., 2011). As oleamide is structurally similar to an endogenous fatty acid amide, anandamide, it has been speculated that oleamide possesses full agonist activity on the cannabinoid (CB_1_) receptor (Boger et al., 2000a; Leggett et al., 2004). On the other hand, other reports have indicated that oleamide has negligible or no effects on the CB_1_ receptor (Mechoulam et al., 1997; Lichtman et al., 2002). Other neuronal receptor systems have also been reported to be associated with the actions of oleamide. Oleamide has shown to inhibit gap junction (connexin)-mediated cell–cell communication (Guan et al., 1997; Boger et al., 1998), and to modulate ionotropic γ-amino butyric acid (GABA_A_) receptors (Verdon et al., 2000), and serotonergic 5-HT_1_, 5-HT_2A/2C_, and 5-HT_7_ receptors (Huidobro-Toro and Harris, 1996; Mueller and Driscoll, 2009). However, little is known about the neuroprotective effect of exogenous oleamide against neuronal death or its underlying intracellular mechanisms.

Kainic acid (KA) has been used to produce an epileptic animal model that mimics human temporal lobe epilepsy (Nadler, 1981) and induce sustained neuronal depolarization and hyperexcitability (Frerking et al., 1998), leading to excitotoxicity in various brain regions including striatum, hippocampus, cerebral cortex, amygdala, and nucleus accumbens, etc. (Zaczek et al., 1980; McGeer and Zhu, 1990; Ferrer et al., 1995; Araujo et al., 2008). Excitotoxicity is a major mechanism of neuronal death in acute brain injury such as stroke, epilepsy, and traumatic brain injury, and is also related to chronic neuronal degenerative diseases including Alzheimer’s disease, Parkinson’s disease, amyotrophic lateral sclerosis, and others (Lau and Tymianski, 2010). As glutamate is the major excitatory neurotransmitter in the CNS, overstimulation of its receptors increases intracellular Ca^2+^ levels by directly opening post-synaptic ion channels and secondarily affecting Ca^2+^ homeostatic mechanisms (Chen et al., 2000). An increase in the cytoplasmic Ca^2+^ level activates a number of Ca^2+^-dependent proteases, particularly calpains, leading to various types of cell death (Araujo et al., 2010) that include necrotic cell death through various different pathways, such as those involving membrane breakdown, cytoskeletal alterations, and nitric-oxide-derived free radicals (Norenberg and Rao, 2007; Mehta et al., 2013), and also triggers apoptosis (Mattson, 2007; Quintanar et al., 2012) or autophagic neuronal death (Kim et al., 2009). Therefore, interest in calpain inhibitors has been growing in an effort to overcome the calpain activity related to cell death that plays a main role in such a wide variety of CNS disorders. The high concentration and activity of calpain that accompanies neuronal degeneration has been seen in the brains of epileptic animal models (Bi et al., 1997; Feng et al., 2011), and KA-induced excitotoxic injury also appear to be prevented by calpain inhibitors (Fitzpatrick et al., 1992).

The present study investigates the antiepileptic and neuroprotective effects of oleamide through calpain inhibition as a potential intracellular mechanism. The ability of oleamide to inhibit calpain activity was demonstrated in both brain tissues of KA-induced *in vivo* epileptic rat models and *in vitro* neuronal systems.

## Materials and Methods

### Chemicals and Reagents

Oleamide, KA, carbamazepine, calpeptin, cresyl violet, hematoxylin and eosin were purchased from Sigma–Aldrich (St. Louis, MO, United States). Fluoro-jade B was purchased from Histo-Chem Inc. (Jefferson, AR, United States). E64d [2S,3S-*trans*-(ethoxycarbonyloxirane-2-carbonyl)-L-leucine-(3-methyl butyl) amide] was obtained from Enzo Life Sciences, Inc. (Farmingdale, NY, United States). The μ-calpain was purchased from Calbiochem (Darmstadt, Germany). Oleamide was suspended in 0.2% methyl cellulose.

### Animals, Surgery, and Drug Administration

Sprague Dawley rats (230–240 g body weight) were purchased from Orient Bio Department (Kyungki-do, Korea). The animals were housed individually in a temperature- (20 ± 1°C) and relative humidity-controlled environment and maintained on a 12-h light/12-h dark cycle. All animal experiments were conducted according to ethical procedures and approved by the Institutional Animal Care and Use Committee of Ewha Womans University (Approval No. Ewha-IACUC 2013-01-041).

For surgery, rats were anesthetized with zoletil (20 mg/kg) and xylazine (9.5 mg/kg) and placed in a stereotaxic apparatus. A Hamilton syringe was used with a mini-pump (Nanometer Injector Syringe Pump; Harvard Apparatus, Holliston, MA, United States) to inject the rats with KA (5 nmole, 0.5 μl) or vehicle (saline, 0.5 μl) intrastriataly at coordinates of 1.2 mm posterior, ± 2.5 mm lateral, and ± 5.5 mm ventral, relative to the bregma. Rats were sacrificed 5 days after surgery. Oleamide (0.5, 2, and 10 mg/kg, p.o.) or vehicle (0.2% methyl cellulose) was orally administered 30 min before the surgery, and administered daily for 4 days after the surgery.

### Monitoring Behavioral Seizures Induced by Intrastriatal Injection of KA

Rats were continuously observed throughout the 3-h period by observers blinded to the treatment. Behavioral seizures were monitored and scored starting from 60 min through 180 min post-surgery. The seizure counts and score were recorded every 10 min to produce representative counts of seizure expression during that period. The convulsive behavior scale consisted of the following seven stages: stage 0, normal behavior; stage 1, wet dog shakes and mouth or facial movements; stage 2, head nodding; stage 3, forelimb clonus; stage 4, rearing; stage 5, rearing and falling; stage 6, tonic seizure or death (Racine, 1972; Sperk et al., 1985).

### Sample Preparation and Western Blotting

Striatal tissues were collected in cold lysis buffer containing 1% Triton X-100, 1 mM EDTA in phosphate-buffered saline (PBS), protease inhibitor cocktail, homogenized, and centrifuged at 10,000 × *g* for 10 min at 4°C. Protein concentration was determined using a BCATM protein assay kit (Thermo Fisher Scientific, Waltham, MA, United States) and assessed by Western blotting. Equal aliquots of the samples were denatured at 100°C, separated by sodium dodecyl sulfate (SDS)-polyacrylamide gel electrophoresis, and blotted onto polyvinylidene fluoride (PVDF) membranes (Millipore Corporation, Billerica, MA, United States). Membranes were incubated in a blocking buffer containing 5% BSA in TTBS for 1-h at room temperature. Immunodetection was performed by incubating membrane blots overnight at 4°C separately with the following primary antibodies (1:1000): anti-CRMP-2 (IBL, Gunma, TS, Japan), anti-Cdk5-p35/25, anti-calpastatin, and anti-synapsin-II (Cell Signaling, Dallas, TX, United States). For chemiluminescent detection, membrane blots were incubated with the horseradish peroxidase (HRP)-conjugated secondary antibody (1:2000) for 2-h at room temperature. Data collection and processing of the integrated optical density of the bands were performed with a luminescent image analyzer (LAS-3000) and IMAGE GAUSE software (Fuji Photo Film, Japan).

### Histological Analysis

Cresyl violet and hematoxylin/eosin (H&E) staining were used to stain tissue sections for histological examination and measurement of neuronal loss. Rats (*n* = 3 per group) were anesthetized with zoletil (20 mg/kg) and xylazine (9.5 mg/kg) and transcardially perfused with PBS followed by 4% paraformaldehyde in PBS. Perfused brains were post-fixed in 4% paraformaldehyde in PBS overnight and subjected to increasing concentrations of alcohol overnight. The brain tissue blocks were embedded in paraffin and the paraffin blocks were cut into a series of 5-μm-thick slices and stained with 0.1% cresyl violet and H&E. All sections were coverslipped with Permount (Fisher Scientific, Fair Lawn, NJ, United States) and were examined with a light microscope (Carl Zeiss, Gottingen, Germany), and photographs were taken with an AxioCam HRC digital camera (Carl Zeiss, Gottingen, Germany).

Fluoro-Jade B (FJB) staining was used to identify degenerating neurons. Briefly, the slides were immersed in 100% ethanol for 3 min, followed by 70% ethanol for 2 min and distilled water for 2 min. The slides were then transferred to 0.06% potassium permanganate for 15 min and gently agitated. After rinsing in distilled water for 2 min, the slides were incubated for 30 min in 0.001% FJB, which was freshly prepared by adding 20 ml of a 0.01% stock FJB solution to 180 ml of 0.1% acetic acid with gentle shaking in the dark. After rinsing three times for 1 min in distilled water, the slides were dried, dehydrated in xylene, and coverslipped.

### *In Vitro* μ-Calpain Assay

There are two prototypical calpain forms, m-calpain, and μ-calpain. The m-calpain, composed of the catalytic subunit calpain II, is located at the membrane and requires 0.2–0.8 mM concentrations of Ca^2+^ for activation. In contrast, μ-calpain, composed of the catalytic subunit calpain II, is located in the cytosol or near the membrane and is activated by 2–80 μM concentrations of Ca^2+^ *in vitro*. Therefore, μ-calpain activity can be affected by minute changes Ca^2+^ concentration. The direct inhibitory effect of oleamide was examined by analyzing μ-calpain activity in calpain 1 (CAPN1)-overexpressing neuronal cells (Lee et al., 2013). In order to measure the μ-calpain inhibitory activity of compounds in SH-SY5Y human neuroblastoma cells, the human CAPN1 gene, which encodes the μ-calpain catalytic subunit, was synthesized (Camins et al., 2006). SH-SY5Y human neuroblastoma cells (ATCC CRL-2266) were cultured in Dulbecco’s Modified Eagle’s Medium containing 10% fetal bovine serum, 100 units/ml penicillin, and 100 μg/ml streptomycin. All cells were maintained at 37°C in humidified conditions under 5% CO_2_. The medium was changed twice weekly, and cultures were split in a 1:5 ratio weekly. For experiments, SH-SY5Y cells were plated in 6-well plates for 48 h. The medium was then removed and replaced with fresh medium without serum, and the cells were maintained for the time periods indicated.

SH-SY5Y cells were transiently transfected with 3 μg of CAPN1-encoding pcDNA3.1/His A expressing the catalytic domain of μ-calpain. After transfection, cells were incubated with each compound in reaction buffer. The cleavage product of pep1 by μ-calpain was measured with the microplate fluorescence reader (SpectraMax Gemini EM, Molecular Devices, Sunnyvale, CA, United States) in kinetic mode at 37°C for 210 min. Fluorescence was measured at 320 nm (ex)/420 nm (em).

### Calpain Substrate Cleavage Assay in *in Vitro* μ-Calpain-Treated Striatal Tissue Extracts

Normal rat striatal tissue lysates (20 μg) were incubated with μ-calpain (0.0125 U/ug) in the presence of 1 mM CaCl_2_ for 30 min at 37°C. Independently, oleamide (30, 100 μM) was co-incubated in the same conditions to identify that the cleavage of collapsin response mediator protein-2 (CRMP-2) and cyclin-dependent kinase-5 (Cdk5)-p35 were mediated by calpain. Western blotting was performed for detection of CRMP-2 and Cdk5-p35.

### Statistical Analysis

Statistical analyses were performed with the Newman–Keuls test and analyses of variance (one-way ANOVA) with GraphPad Prism version 5.0d (GraphPad Software, Inc., La Jolla, San Diego, CA, United States). The *p*-value < 0.05 was considered statistically significant. All results were expressed as the mean ± SEM of at least three independent experiments.

## Results

### Kainic Acid (KA)-Induced Behavioral Seizure Activity and Antagonizing Effect of Oleamide

**FIGURE 1:**
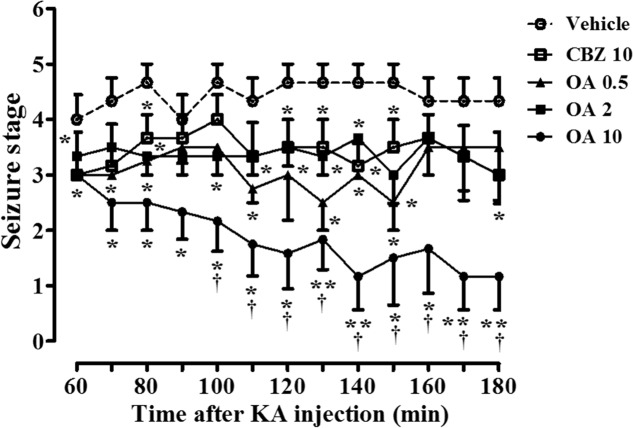
Oleamide (OA) reduces KA-induced behavioral seizure severity throughout the 3-h rating period, and is comparable to carbamazepine (CBZ). Seizures were initiated in rats by intrastriatal injection of KA (5 nmol) into the right striatum (the left striatum was treated with saline). Rats were orally administered either vehicle (0.2% methyl cellulose), OA (0.5, 2, and 10 mg/kg), or CBZ (10 mg/kg) 30 min before the KA injection. Behavioral seizures were monitored by blinded raters for 3-h after the surgery and scored for 2-h from 60 to 180 min. The data are the means ± SEM of 4–6 independent experiments. ^∗^*p* < 0.01 and ^∗∗^*p* < 0.001 (ANOVA) compared with KA-only data (vehicle). ^†^*p* < 0.05 vs. CBZ-treated data.

Systemic (intraperitoneal) administration of KA in rodents induced epileptic seizures similar to human temporal lobe epilepsy, with spontaneous seizures, as well as seizure-induced neuronal cell death (Ben-Ari, 1985; Bortolatto et al., 2011). A clinically used prototype antiepileptic drug, carbamazepine, has shown to produce the dose-dependent protective effects on spontaneous seizures in rats with KA-induced epilepsy by intraperitoneal injection at concentration ranges of 30–100 mg/kg (Grabenstatter et al., 2007), and the protection against pentylenetetrazole-induced seizure by oral administration at 10–20 mg/kg (Reeta et al., 2011). We first examined whether oleamide inhibits KA-induced epileptic behavior. Rats were pretreated with an oral injection of oleamide (0.5, 2, or 10 mg/kg suspended in 0.2% methylcellulose or vehicle (0.2% methylcellulose) 30 min prior to intrastriatal infusion of KA (5 nmole/0.5 μl). **Figure 1** shows the time course of behavioral seizures in each treatment group. In the vehicle group, rats showed extreme seizures behaviors. Maximum average Racine’s score reached 4.4 points (Racine, 1972). Oleamide (0.5, 2, 10 mg/kg) produced anticonvulsive effects on KA-induced behavioral seizures in a dose-dependent manner. Especially, oleamide at 10 mg/kg dramatically reduced the seizure scores (from 4.3 to 1.2 at 180 min) that were significantly greater than that of same concentration (10 mg/kg) of carbamazepine (3.0 at the same time point).

### Protective Effects of Oleamide against KA-Induced Neuronal Damage in Striatum

It has been shown that KA induces internucleosomal DNA fragmentation and loss of striatal neurons (Wang et al., 2008). We examined whether oleamide is protective against KA-induced excitotoxic neuronal damage, using histological staining analyses. Cresyl violet and hematoxylin/eosin (H&E) staining data clearly showed the KA-induced neuronal damage instriatal brain tissue sections, which is similar to other reports that KA administration resulted in the presence of mainly pyknotic nuclei (Nash et al., 1991; Liao et al., 2016).

**FIGURE 2:**
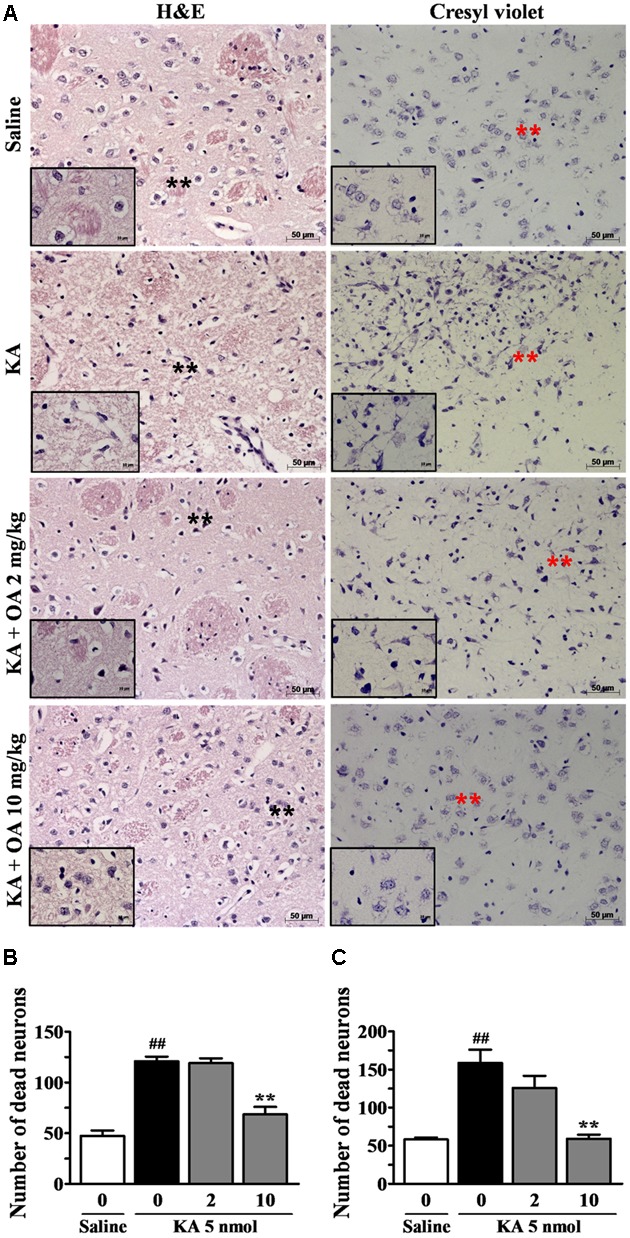
Protective effects of oleamide (OA) on KA-induced neuronal damage. Brains were coronally sectioned into 5 μm sections 5 days after the stereotaxic injection of saline and KA 5 nmol. The coronal sections were mounted on slides and stained with H&E and cresyl violet **(A)**. Asterisks indicate the tissue areas that are magnified in the smaller panels. Striatal regions were enlarged ×200 and ×630. Scale bars = 50 and 10 μm. **(B,C)** The number of damaged neuronal cells per brain (*n* = 3) section was quantitated using the Image J program. ##*p* < 0.001 vs. saline, and ^∗∗^*p* < 0.001 vs. vehicle.

H&E staining showed that striatal tissues from rats exposed to KA (5 nmole) exhibited extensive cell loss and pyknotic nuclei at the neuropil. In contrast, rats pretreated with oleamide prior to KA injection showed a significant reduction in pyknotic nuclei, while pyknotic nuclei remained in cells of vehicle-treated KA-epileptic rat striatal tissues. In addition, the protective effect of oleamide was also observed in cresyl violet staining, which enables visualization of neuronal damage and cell dispersion (**Figure 2A**). The number of damaged neuronal cells was measured by counting cells in each of two fields randomly selected from each of three separate striatal tissue sections of three rats per group. The general criteria used to score damaged cells included the number of hyperchromatic nuclei and cytoplasmic vacuolation. Quantitative analyses (**Figures 2B,C**) revealed a dramatic protective effect of oleamide (10 mg/kg) following KA administration in the striatum, as assessed by both cresyl violet staining (*p* < 0.001) and H&E staining (*p* < 0.001), as compared with KA alone.

**FIGURE 3:**
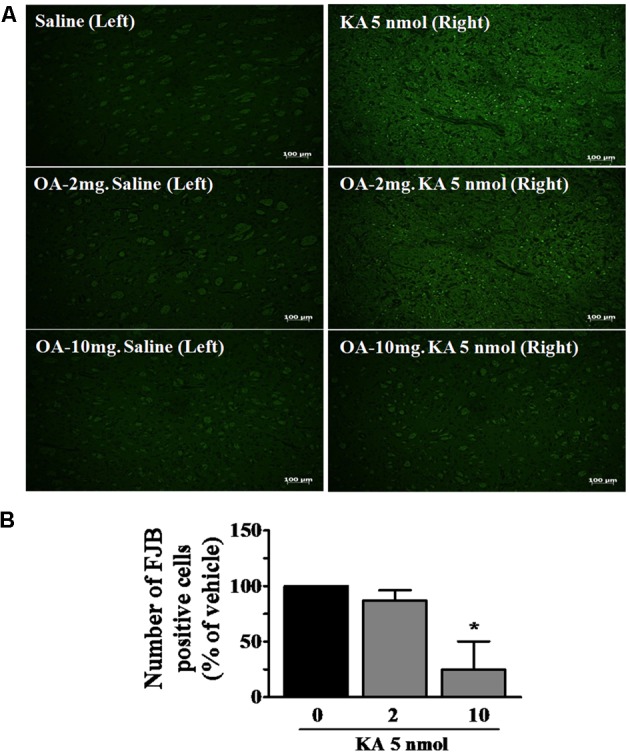
Neuroprotective effects of oleamide (OA) against KA-induced neuronal cell death. Fluoro-jade B (FJB) staining was performed on coronally sectioned rat brains to identify damaged neurons 5 days after the induction of KA. **(A)** Representative magnification (100×) photomicrographs showing FJB-positive neurons indicating neurodegeneration in the striatum. Three animals were randomly assigned to each condition. Scale bar = 100 μm. **(B)** FJB-positive neurons were quantitated using the Image J program. ^∗^*p* < 0.05 vs. vehicle.

The protective effects of oleamide against neuronal damage were further confirmed using staining with FJB, which specifically stains dying neurons and becomes fluorescent green. FJB is an anionic fluoresce in derivative useful for histological staining of neurons undergoing cellular degeneration (Schmued and Hopkins, 2000; Miyamoto et al., 2008; Furtado et al., 2011). Numerous FJB-positive cells were observed in the striatum following KA (5 nmole) injection, as similarly seen in the hippocampus by other report (Miyamoto et al., 2008). In the oleamide-treated animals, only a few positive cells were found in the striatum, and the effect was dose-dependent (**Figure 3A**). No FJB-positive cells were detected in the left striatum of sham-operated and oleamide-treated control rat brains. Quantification of FJB-stained cells in the region revealed a statistically significant decrease in the number of stained cells following oleamide (10 mg/kg) administration (**Figure 3B**). FJB staining data provide further evidence confirming the protective effects of oleamide against KA-induced excitotoxic brain damage.

### Inhibitory Effect of Oleamide on KA-Induced Calpain Activation in Epileptic Rat Striatal Tissue

**FIGURE 4:**
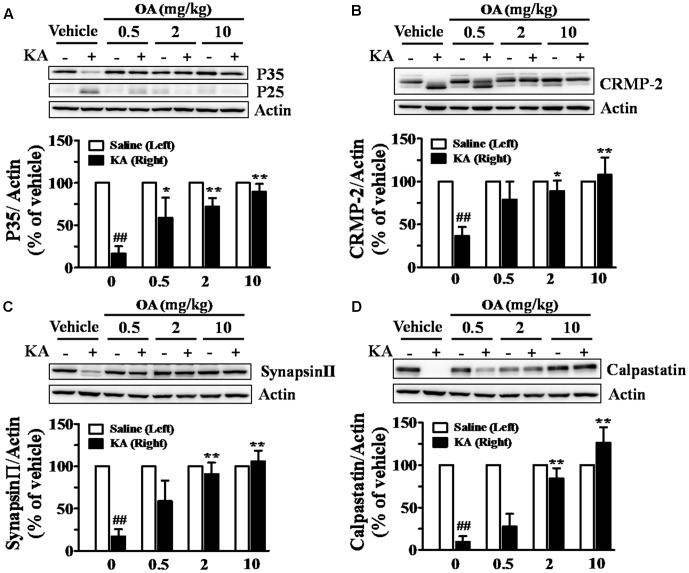
Inhibitory effects of oleamide (OA) on KA-induced calpain activation in rat striatum. Rats were administered OA (0.5, 2, and 10 mg/kg, p.o.) or vehicle (0.2% methyl cellulose) 30 min before intrastriatal injection of KA (5 nmol), whereas the left striatum was treated with saline only, and given repeated oral doses of OA daily for the next 4 days. Striatal lysates were subjected to Western blot analysis using anti-Cdk5-p35 **(A)**, anti-CRMP-2 **(B)**, anti-synapsin-II **(C)**, and anti-calpastatin **(D)**. Data are the means ± SEM of 3–7 experiments. ^##^*p* < 0.001 vs. vehicle, ^∗^*p* < 0.01 and ^∗∗^*p* < 0.001 vs. KA-treated vehicle.

It was shown that injection of KA into the striatum increased calpain mRNA (Campbell et al., 2004). Calpain enzyme has been shown to play a central role in KA-induced excitotoxicity by cleaving a large number of substrates, including a Cdk5 coactivator (p35) and CRMPs (Bevers and Neumar, 2008). KA-induced calpain-specific Cdk5-p35/25 pathway activation, represented by the conversion of Cdk5-p35 to p25, was also blocked by pretreatment with oleamide (**Figure 4A**). It was previously shown that CRMP-2 (∼62 kDa) is specifically cleaved into a 58 kDa protein under ischemic conditions and that CRMP-2 cleavage is mediated by calpain (Chung et al., 2005). The oleamide-induced change in cleavage patterns of those substrates was examined in striatal tissues of KA-induced epileptic rats. Oral administration of oleamide (0.5–10 mg/kg) antagonized KA-induced cleavage of CRMP-2, as reflected by a dose-dependent increase in the level of uncleaved 62 kDa CRMP-2 and a decrease in the concentration of 58 kDa CRMP-2 (**Figure 4B**). Synapsin-II protein is a marker of synaptic activity and plasticity (Zurmohle et al., 1996; Ferreira et al., 1998) and its level has been shown to be reduced in epileptic or neuronal death conditions (Karanian et al., 2007). We observed that the level of synapsin-II was markedly decreased in KA-induced epileptic rat striatal tissues, and that oleamide prevented the KA-induced reduction of synapsin-II to control levels in the normal rat striatum (**Figure 4C**). Our data suggest a protective effect of oleamide against KA-induced disruptions in synaptic integrity. In addition, oleamide protected the expression of an endogenous calpain inhibitory protein, calpastatin, the level of which was reduced by KA (**Figure 4D**).

### Calpain Inhibitory Effects of Oleamide in *in Vitro* μ-Calpain-Treated Striatal Tissue Extracts and in Calpain 1 (CAPN1)-Overexpressed Neuronal Cells

**FIGURE 5:**
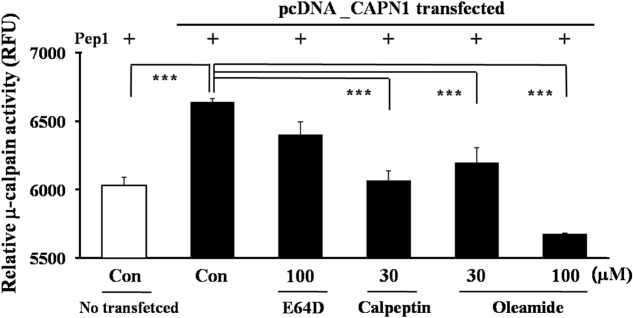
Inhibition of calpain activity by oleamide (OA) in μ-calpain overexpressed SH-SY5Y cells. μ-Calpain (CAPN1)-overexpressed SH-SY5Y cells were incubated with substrate pep 1 (peptide derived from the Cdk5-p35 cleavage site) and compounds. The relative change in fluorescence induced by μ-calpain-mediated pep1 cleavage was quantified. Fluorescence was measured at 320 nm (ex) and 420 nm (em). ^∗∗∗^*p* < 0.001 vs. μ-calpain-transfected control.

The cell-based μ-calpain assay was performed to investigate the μ-calpain inhibitory activity of oleamide in SH-SY5Y cells. μ-Calpain is endogenously expressed in SH-SY5Y cells, but its activity is not potent enough to sufficiently cleave the exogenous substrate pep1. Therefore, the large subunit of μ-calpain, CAPN1, was additionally transfected to visibly measure the compound μ-calpain inhibitory activity in SH-SY5Y cells. E64D, an irreversible calpain inhibitor, was prepared by esterification of the free carboxylic acid group of E64C to improve cell permeability. The results showed that μ-calpain activity was highest in CAPN1-transfected cells. Treatment with oleamide (30, 100 μM) reduced μ-calpain activity with better potency than did E64D (100 μM). Oleamide (30 μM) produced an inhibitory effect to a similar degree as calpeptin (30 μM), and the higher concentration of oleamide (100 μM) significantly decreased the enhanced μ-calpain activity to the level of control cells (**Figure 5**). This result confirms the direct calpain inhibitory effect of oleamide, suggesting the possibility that it can be used as a novel calpain inhibitor.

**FIGURE 6:**
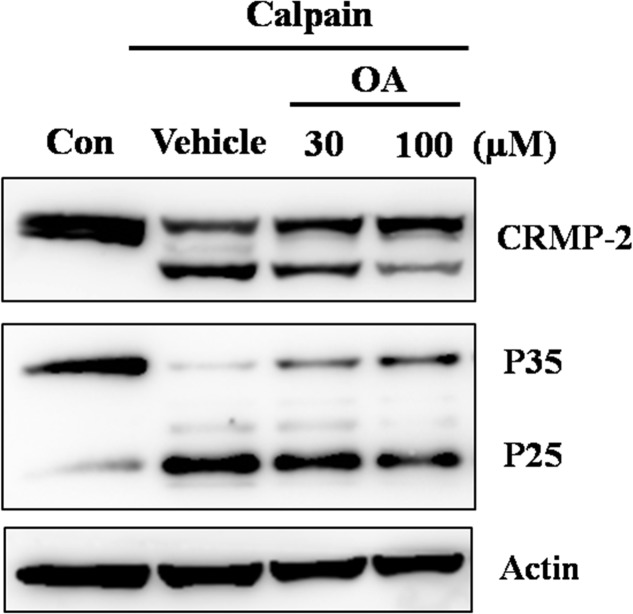
Inhibition of calpain activity by oleamide in rat striatum tissue lysates. Rat striatum tissue lysates (20 μg) were incubated with μ-calpain (0.0125 U/ug) in the presence of 1 mM CaCl_2_ for 30 min at 37°C. OA (30, 100 μM) was co-incubated in the same conditions to assess whether the cleavage of CRMP-2 and Cdk5-p35 were mediated by calpain. Western blotting was performed to detect cleavage of CRMP-2 and Cdk5-p35.

The *in vitro* calpain-inhibitory effect of oleamide was further confirmed by adding oleamide to rat striatal tissue extracts incubated with purified μ-calpain. As shown in **Figure 6**, oleamide (30, 100 μM) remarkably decreased the calpain-induced cleavage of both CRMP-2 and Cdk5-p35. These results suggest the possibility that oleamide is a novel calpain inhibitor.

## Discussion

The present study investigates the *in vivo* antiepileptic and neuroprotective effects of exogenous oleamide against KA-induced excitotoxic brain damage, and asks whether calpain inhibition is an intracellular mechanism of those effects. We demonstrated the preventive effects of oleamide against epileptic behavior in KA-induced epileptic rat model and against excitotoxicity-induced calpain activation that leads to neuronal death. The ability of oleamide to inhibit calpain activity was examined in both brain tissue of a KA-induced epileptic rat model and cultured neurons.

Oleamide is an endogenous fatty acid amide and shares a structure and some characteristics with the endocannabinoid anandamide. Both oleamide and anandamide are degraded by fatty acid amide hydrolase (Boger et al., 2000a). While many biological effects of oleamide are documented, its molecular and signaling mechanisms are less well defined than those of cannabinoids. The endocannabinoid system has been identified as a potential target for the treatment of several disorders of the CNS, including epilepsy and excitotoxicity (Marsicano et al., 2003; Monory et al., 2006). An increasing number of studies have implicated the endogenous fatty acid amide system as a new target for neuronal damage, mainly studies on anandamide and fatty acid amide hydrolase inhibitors (Karanian et al., 2007; Naidoo et al., 2012). Several studies have shown that endocannabinoid deficiency may contribute to the pathophysiology of chronic pain including migraine and inflammatory pain (Lastres-Becker et al., 2002; Clapper et al., 2010; Nozaki et al., 2015). The novel fatty acid amide hydrolase inhibitor, URB-597, has been demonstrated to produce anticonvulsant effect on seizures induced by pentylenetetrazole in rats (Vilela et al., 2013), and to block neuronal hyperactivity in neurons (Nozaki et al., 2015), which can be considered a similar mechanism to that seen in epilepsy. Interestingly, the administration of an endocannabinoid uptake inhibitor (AM404) that kept the fatty acid amide levels high to the rats injected intrastriatally with 3-nitropropionic acid, a toxin that selectively damages striatal GABAergic neurons, was shown to attenuate hyperactive motor disturbances (Lastres-Becker et al., 2002). These reports suggest that fatty acid amides can play important roles as signaling molecules that are involved in mechanisms for epilepsy and motor hyperactivity. Another fatty acid amide hydrolase inhibitor (AM374) that was reported to cause a prolonged elevation of the anandamide level in the brain reduced KA-induced seizures by promoting CB_1_ receptor signaling (Karanian et al., 2007; Shubina et al., 2015). Thus, fatty acid amide hydrolase inhibitors have been suggested to be relevant to the excitotoxic protection by enhancement of the endocannabinoid system, which is elicited via inhibition of endocannabinoid degradation.

Although oleamide is an endogenous fatty acid amide, which is degraded by fatty acid amide hydrolase, only a few of studies have reported on the *in vivo* antiepileptic and neuroprotective effects by exogenously administered oleamide. We first demonstrated that oral administration of oleamide significantly reduced seizure behavior in KA-induced epileptic rats. The KA-induced chronic epileptic animal model has been known to mimic human temporal lobe epilepsy and status epilepticus (Nadler, 1981). Many reports have shown that changes in the hippocampus and/or cortex are mainly involved in epileptic seizures. The striatum, however, is also well reported as an important brain region of the neuronal damage with relation to the occurrence of convulsions, including KA-induced seizures in rats (Pisa et al., 1980). In rats induced epilepsy by injection of KA into the striatum, anti-seizure effect of oleamide was more potent than that of carbamazepine, a clinically long-used anticonvulsant agent (**Figure 1**). Previously, the anticonvulsant effect of oleamide was only shown to produce against the behavioral seizures induced by pentylenetetrazole, the most commonly used acute seizure model (Wu et al., 2003; Solomonia et al., 2008). According to the Wu et al.’s (2003) report, intraperitoneal administration of oleamide (43.8–700 mg/kg) to mice significantly attenuated the latency of seizure onset induced by pentylenetetrazole (85 mg/kg, i.p.) that was administered 30 min after the injection of oleamide, but produced no effect on seizures induced by other convulsive agents, such as picrotoxin, semicarbazide, strychnine, and caffeine. This report does not fully explain the selective action of oleamide on pentylenetetrazole-induced seizures, and the *in vivo* dose range of oleamide used in the report is exceptionally higher than that reported by other researchers. In another short report, oleamide in a dose of 10 mg/kg injected (i.p.) to rats was shown to produce anti-seizure effect specifically on the degree (severity) of convulsions, but not decrease the duration and latency of convulsions (Solomonia et al., 2008).

In addition, we found that oleamide prevents KA-induced neuronal death through histological analyses of cellular integrity in striatal sections of KA-induced epileptic rats (**Figures 2**, **3**). The striatum has been demonstrated to express a large number of binding sites for all classes of glutamate receptors (Albin et al., 1992; Wullner et al., 1994; Miyamoto et al., 2008), and the intrastriatal injection of KA has been to lead to substantial neuronal loss in striatal tissues (Wang et al., 2006). Apoptotic and necrotic death of neurons is involved in KA-induced excitotoxicity *in vivo* (Wang et al., 2005; Vincent and Mulle, 2009). The KA-induced striatal damage observed in our demonstrated experimental condition can be considered from direct toxic effect of KA, presumably through excessive stimulation of striatal glutamate receptors, since the neuronal damage was observed in only right side of striatum where KA was directly injected, but not in saline-injected left side of striatum. Excessive stimulation of glutamate receptors and neuronal damage due to KA is thought to be a result of a large influx of Ca^2+^ into neurons and by a dysfunction of downstream signaling systems (Koh et al., 1990; Lipton and Rosenberg, 1994) including calpain activation.

It has been reported that neuronal damage can spread from the KA-injected striatum into contiguous structures (Zaczek et al., 1980), and can induce locomotor changes and epileptogenesis (McGeer and Zhu, 1990). Although KA-induced seizures are followed by extensive neurodegenerative changes, the seizure-induced brain damage is still debated (not conclusively demonstrated) both in humans and in animal models of epilepsy (Rossini et al., 2017).

Alterations in Ca^2+^ homeostasis lead to persistent, pathologic overactivation of calpain in a number of neurodegenerative diseases (Vosler et al., 2008). Initially, calpain activation was thought to cause only necrotic cell death, while activation of caspase led to programmed cell death. There is a shared mechanism of calpain activation in neurodegenerative diseases, such as epilepsy, Alzheimer’s disease, and Parkinson’s disease (Lau and Tymianski, 2010; Lopatniuk and Witkowski, 2011). In particular, calpain-specific cleavage of Cdk5-p35 to p25 has been implicated in the neurological damage seen in many neurological disorders (Kanungo et al., 2009; Cheung and Ip, 2012). Calpains have long been implicated in neuronal cell death induced by triggers of neuronal injury including excitotoxicity. Putkonen et al.’s (2011) reported that KA produced a dose-dependent increase in intracellular Ca^2+^concentration and raised calpain activity, followed by induction of phosphorylation of Cdk5 and cleavage of Cdk-p35, which are believed to be involved in KA-mediated degeneration of glutamatergic synapses in the rat hippocampus (Putkonen et al., 2011). Also in the striatum, it has been shown that intrastriatal injection of KA increases calpain mRNA (Campbell et al., 2004). In addition, calpain activation is involved in manganese-induced neuronal death in rat striatum (Quintanar et al., 2012).

Since many calpain inhibitors have been shown to produce antiepileptic effects (Lubisch et al., 2000), we postulated that oleamide can also affect calpain activity, and investigated cleavage of the representative calpain substrate protein, Cdk-p35 in KA-induced epileptic rat striatal tissues. We found that the KA-induced enhancement of Cdk5-p35 cleavage to p25 was significantly blocked by oral administration of oleamide (**Figure 4A**), implying that oleamide could produce antiepileptic and neuroprotective effects by the mechanism related to calpain inhibition.

The KA-induced significant neuronal loss can lead to the axonal degeneration that is preceded by disruption of Ca^2+^ homeostasis, causing calpain activation and the proteolytic degradation of axonal proteins (Saatman et al., 1996). CRMP-2 is known to be involved in neuronal differentiation and control of neuronal polarity and axonal outgrowth (Yoshimura et al., 2006). In addition, more recent studies have reported that CRMP-2 can bind to the voltage-gated calcium channel Cav2.2, and this interaction may play a crucial role in neurotransmitter release from the presynaptic terminals of hippocampal neurons (Wang et al., 2010). In our previous study, CRMP-2 protein was shown to be altered into a cleaved form with a size of 58 kDa in the brain under ischemic conditions (Chung et al., 2005), which was later shown to be induced by calpain activation (Hou et al., 2009). Since then, it has been discovered that CRMPs are cleaved in severe neurodegenerative conditions and their expression levels are changed in several neuronal diseases. CRMP-2 was reduced in patients with temporal lobe epilepsy (Czech et al., 2004). A relatively new antiepileptic drug, lacosamide, was reported to modulate CRMP-2 and inactivate voltage-gated sodium channels, which were identified as its antiepileptic mechanisms (Saussele, 2008; Patyar and Medhi, 2010). In the present study, calpain-induced CRMP-2 cleavage was observed in KA-induced epileptic rat striatal extracts, and, similar to Cdk5-p35, oleamide significantly blocked CRMP-2 cleavage (**Figure 4B**). Additional evidence implying that oleamide can improve KA-induced synaptic dysfunction was provided by measuring the changes in the level of synapsin-II, one of the synaptic marker proteins. KA dramatically reduced the level of synapsin-II, and oleamide dose-dependently protected the synapsin-II levels (**Figure 4C**). These data indicate that oleamide can block the calpain-mediated cleavage of substrate proteins that play important roles in the regulation of neuronal survival/death and neuronal activity.

We further confirmed the calpain inhibitory effects of oleamide by examining changes in endogenous calpastatin levels. An endogenous calpain inhibitory protein, calpastatin, has been proposed to play pro-survival roles in adult neurons under degenerative conditions, including in models of ischemia–excitotoxicity (Yang et al., 2013). Recently, exogenous transgenic expression of calpastatin was used to provide definitive evidence for calpain’s involvement in sciatic and optic nerve degeneration after sectioning *in vivo* (Ma et al., 2013). Data (**Figure 4D**) showing the ability of oleamide to enhance the level of endogenous calpastatin, which is reduced in KA-induced epileptic rat brain, suggest that its antiepileptic and possible neuroprotective effects are due to calpain inhibition.

So far, oleamide was assumed to exert its various biological actions through as-yet undefined membrane receptors. The calpain inhibitory effect of oleamide has not been reported previously. We hypothesized that oleamide might play a role as a calpain inhibitor. To investigate this, *in vitro* experiments were performed. In calpain-transfected cells, oleamide was observed to decrease the μ-calpain activity more effectively than the known calpain inhibitors E64d and calpeptin (**Figure 5**). In addition, the CRMP-2 and Cdk5 degradation observed in rat brain tissue lysates directly incubated with μ-calpain were inhibited by exogenous treatment with oleamide (**Figure 6**). These results provide evidence for the possibility of direct calpain inhibition by oleamide. It can be speculated that oleamide may enter into the intracellular space through an endogenous fatty acid amide membrane transporter that has been shown to transport anandamide (Mechoulam and Deutsch, 2005), and then inhibit calpain directly and/or indirectly, however, evidence for this theory is still lacking.

Taken together, our findings reveal that exogenous oleamide attenuates not only KA-induced behavioral seizures but also calpain-mediated neuronal cell death in the brain, suggesting that oleamide is a novel promising drug candidate for various neuronal diseases including epilepsy. In addition, the antiepileptic and neuroprotective effects of oleamide could be mediated through a novel mechanism by calpain inhibition, although its direct calpain inhibitory effect needs to be further pursued.

## Author Contributions

HYN performed experiments, analyzed the data, and wrote the first draft of manuscript. EJN, EL, and YK analyzed data and involved in the manuscript preparation. H-JK designed and supervised the research, analyzed data, and wrote the manuscript. All authors read and approved the final manuscript.

## Conflict of Interest Statement

The authors declare that the research was conducted in the absence of any commercial or financial relationships that could be construed as a potential conflict of interest.

## References

[B1] Akanmu M. A. Adeosun S. O. Ilesanmi O. R. (2007). Neuropharmacological effects of oleamide in male and female mice. *Behav. Brain Res.* 182 88–94. 10.1016/j.bbr.2007.05.006 17588682

[B2] Albin R. L. Makowiec R. L. Hollingsworth Z. R. Dure L. S. I. V. Penney J. B. Young A. B. (1992). Excitatory amino acid binding sites in the basal ganglia of the rat: a quantitative autoradiographic study. *Neuroscience* 46 35–48. 10.1016/0306-4522(92)90006-N 1317515

[B3] Ano Y. Ozawa M. Kutsukake T. Sugiyama S. Uchida K. Yoshida A. (2015). Preventive effects of a fermented dairy product against Alzheimer’s disease and identification of a novel oleamide with enhanced microglial phagocytosis and anti-inflammatory activity. *PLOS ONE* 10:e0118512. 10.1371/journal.pone.0118512 25760987 PMC4356537

[B4] Araujo I. M. Carreira B. P. Carvalho C. M. Carvalho A. P. (2010). Calpains and delayed calcium deregulation in excitotoxicity. *Neurochem. Res.* 35 1966–1969. 10.1007/s11064-010-0323-z 21110090

[B5] Araujo I. M. Gil J. M. Carreira B. P. Mohapel P. Petersen A. Pinheiro P. S. (2008). Calpain activation is involved in early caspase-independent neurodegeneration in the hippocampus following status epilepticus. *J. Neurochem.* 105 666–676. 10.1111/j.1471-4159.2007.05181.x 18088374

[B6] Ben-Ari Y. (1985). Limbic seizure and brain damage produced by kainic acid: mechanisms and relevance to human temporal lobe epilepsy. *Neuroscience* 14 375–403. 10.1016/0306-4522(85)90299-4 2859548

[B7] Bevers M. B. Neumar R. W. (2008). Mechanistic role of calpains in postischemic neurodegeneration. *J. Cereb. Blood Flow Metab.* 28 655–673. 10.1038/sj.jcbfm.9600595 18073773

[B8] Bi X. Chen J. Baudry M. (1997). Developmental changes in calpain activity, GluR1 receptors and in the effect of kainic acid treatment in rat brain. *Neuroscience* 81 1123–1135. 10.1016/S0306-4522(97)00218-2 9330373

[B9] Boger D. L. Fecik R. A. Patterson J. E. Miyauchi H. Patricelli M. P. Cravatt B. F. (2000a). Fatty acid amide hydrolase substrate specificity. *Bioorg. Med. Chem. Lett.* 10 2613–2616. 11128635 10.1016/s0960-894x(00)00528-x

[B10] Boger D. L. Patterson J. E. Guan X. Cravatt B. F. Lerner R. A. Gilula N. B. (1998). Chemical requirements for inhibition of gap junction communication by the biologically active lipid oleamide. *Proc. Natl. Acad. Sci. U.S.A.* 95 4810–4815. 10.1073/pnas.95.9.4810 9560184 PMC20169

[B11] Boger D. L. Sato H. Lerner A. E. Hedrick M. P. Fecik R. A. Miyauchi H. (2000b). Exceptionally potent inhibitors of fatty acid amide hydrolase: the enzyme responsible for degradation of endogenous oleamide and anandamide. *Proc. Natl. Acad. Sci. U.S.A.* 97 5044–5049. 10805767 10.1073/pnas.97.10.5044 PMC25778

[B12] Bortolatto C. F. Jesse C. R. Wilhelm E. A. Ribeiro L. R. Rambo L. M. Royes L. F. (2011). Protective effect of 2,2’-dithienyl diselenide on kainic acid-induced neurotoxicity in rat hippocampus. *Neuroscience* 193 300–309. 10.1016/j.neuroscience.2011.07.038 21820494

[B13] Camins A. Verdaguer E. Folch J. Pallas M. (2006). Involvement of calpain activation in neurodegenerative processes. *CNS Drug Rev.* 12 135–148. 10.1111/j.1527-3458.2006.00135.x 16958987 PMC6494133

[B14] Campbell S. J. Finlay M. Clements J. M. Wells G. Miller K. M. Perry V. H. (2004). Reduction of excitotoxicity and associated leukocyte recruitment by a broad-spectrum matrix metalloproteinase inhibitor. *J. Neurochem.* 89 1378–1386. 10.1111/j.1471-4159.2004.02441.x 15189340

[B15] Chen C. J. Liao S. L. Kuo J. S. (2000). Gliotoxic action of glutamate on cultured astrocytes. *J. Neurochem.* 75 1557–1565. 10.1046/j.1471-4159.2000.0751557.x 10987836

[B16] Cheung Z. H. Ip N. Y. (2012). Cdk5: a multifaceted kinase in neurodegenerative diseases. *Trends Cell Biol.* 22 169–175. 10.1016/j.tcb.2011.11.003 22189166

[B17] Chung M. A. Lee J. E. Lee J. Y. Ko M. J. Lee S. T. Kim H. J. (2005). Alteration of collapsin response mediator protein-2 expression in focal ischemic rat brain. *Neuroreport* 16 1647–1653. 10.1097/01.wnr.0000176520.49841.e6 16189471

[B18] Clapper J. R. Moreno-Sanz G. Russo R. Guijarro A. Vacondio F. Duranti A. (2010). Anandamide suppresses pain initiation through a peripheral endocannabinoid mechanism. *Nat. Neurosci.* 13 1265–1270. 10.1038/nn.2632 20852626 PMC3260554

[B19] Cravatt B. F. Prospero-Garcia O. Siuzdak G. Gilula N. B. Henriksen S. J. Boger D. L. (1995). Chemical characterization of a family of brain lipids that induce sleep. *Science* 268 1506–1509. 10.1126/science.7770779 7770779

[B20] Czech T. Yang J. W. Csaszar E. Kappler J. Baumgartner C. Lubec G. (2004). Reduction of hippocampal collapsin response mediated protein-2 in patients with mesial temporal lobe epilepsy. *Neurochem. Res.* 29 2189–2196. 10.1007/s11064-004-7025-3 15672539

[B21] Fedorova I. Hashimoto A. Fecik R. A. Hedrick M. P. Hanus L. O. Boger D. L. (2001). Behavioral evidence for the interaction of oleamide with multiple neurotransmitter systems. *J. Pharmacol. Exp. Ther.* 299 332–342. 11561096

[B22] Feng Z. H. Hao J. Ye L. Dayao C. Yan N. Yan Y. (2011). Overexpression of mu-calpain in the anterior temporal neocortex of patients with intractable epilepsy correlates with clinicopathological characteristics. *Seizure* 20 395–401. 10.1016/j.seizure.2011.01.010 21315622 PMC3600948

[B23] Ferreira A. Chin L. S. Li L. Lanier L. M. Kosik K. S. Greengard P. (1998). Distinct roles of synapsin I and synapsin II during neuronal development. *Mol. Med.* 4 22–28. 9513186 PMC2230269

[B24] Ferrer I. Martin F. Serrano T. Reiriz J. Perez-Navarro E. Alberch J. (1995). Both apoptosis and necrosis occur following intrastriatal administration of excitotoxins. *Acta Neuropathol.* 90 504–510. 10.1007/BF00294812 8560984

[B25] Fitzpatrick J. S. Shahi K. Baudry M. (1992). Effect of seizure activity and calpain inhibitor I on LTP in juvenile hippocampal slices. *Int. J. Dev. Neurosci.* 10 313–319. 10.1016/0736-5748(92)90020-Z 1384274

[B26] Fowler C. J. (2004). Oleamide: a member of the endocannabinoid family? *Br. J. Pharmacol.* 141 195–196. 10.1038/sj.bjp.0705608 14691053 PMC1574195

[B27] Frerking M. Malenka R. C. Nicoll R. A. (1998). Synaptic activation of kainate receptors on hippocampal interneurons. *Nat. Neurosci.* 1 479–486. 10.1038/2194 10196545

[B28] Furtado M. A. Castro O. W. Del Vecchio F. de Oliveira J. A. Garcia-Cairasco N. (2011). Study of spontaneous recurrent seizures and morphological alterations after status epilepticus induced by intrahippocampal injection of pilocarpine. *Epilepsy Behav.* 20 257–266. 10.1016/j.yebeh.2010.11.024 21237720

[B29] Grabenstatter H. L. Clark S. Dudek F. E. (2007). Anticonvulsant effects of carbamazepine on spontaneous seizures in rats with kainate-induced epilepsy: comparison of intraperitoneal injections with drug-in-food protocols. *Epilepsia* 48 2287–2295. 10.1111/j.1528-1167.2007.01263.x 17711461

[B30] Guan X. Cravatt B. F. Ehring G. R. Hall J. E. Boger D. L. Lerner R. A. (1997). The sleep-inducing lipid oleamide deconvolutes gap junction communication and calcium wave transmission in glial cells. *J. Cell Biol.* 139 1785–1792. 10.1083/jcb.139.7.1785 9412472 PMC2132638

[B31] Hou S. T. Jiang S. X. Aylsworth A. Ferguson G. Slinn J. Hu H. (2009). CaMKII phosphorylates collapsin response mediator protein 2 and modulates axonal damage during glutamate excitotoxicity. *J. Neurochem.* 111 870–881. 10.1111/j.1471-4159.2009.06375.x 19735446

[B32] Huidobro-Toro J. P. Harris R. A. (1996). Brain lipids that induce sleep are novel modulators of 5-hydroxytrypamine receptors. *Proc. Natl. Acad. Sci. U.S.A.* 93 8078–8082. 10.1073/pnas.93.15.8078 8755606 PMC38878

[B33] Huitron-Resendiz S. Gombart L. Cravatt B. F. Henriksen S. J. (2001). Effect of oleamide on sleep and its relationship to blood pressure, body temperature, and locomotor activity in rats. *Exp. Neurol.* 172 235–243. 10.1006/exnr.2001.7792 11681856

[B34] Kanungo J. Zheng Y. L. Amin N. D. Pant H. C. (2009). Targeting Cdk5 activity in neuronal degeneration and regeneration. *Cell Mol. Neurobiol.* 29 1073–1080. 10.1007/s10571-009-9410-6 19455415 PMC5603152

[B35] Karanian D. A. Karim S. L. Wood J. T. Williams J. S. Lin S. Makriyannis A. (2007). Endocannabinoid enhancement protects against kainic acid-induced seizures and associated brain damage. *J. Pharmacol. Exp. Ther.* 322 1059–1066. 10.1124/jpet.107.120147 17545313

[B36] Kim H. Choi J. Ryu J. Park S. G. Cho S. Park B. C. (2009). Activation of autophagy during glutamate-induced HT22 cell death. *Biochem. Biophys. Res. Commun.* 388 339–344. 10.1016/j.bbrc.2009.08.007 19665009

[B37] Koh J. Y. Goldberg M. P. Hartley D. M. Choi D. W. (1990). Non-NMDA receptor-mediated neurotoxicity in cortical culture. *J. Neurosci.* 10 693–705. 2406381 10.1523/JNEUROSCI.10-02-00693.1990 PMC6570171

[B38] Lastres-Becker I. Gomez M. De Miguel R. Ramos J. A. Fernandez-Ruiz J. (2002). Loss of cannabinoid CB_1_ receptors in the basal ganglia in the late akinetic phase of rats with experimental Huntington’s disease. *Neurotox. Res.* 4 601–608. 10.1080/10298420290030514 12709298

[B39] Lau A. Tymianski M. (2010). Glutamate receptors, neurotoxicity and neurodegeneration. *Pflugers Arch.* 460 525–542. 10.1007/s00424-010-0809-1 20229265

[B40] Lee E. Eom J. E. Kim H. L. Baek K. H. Jun K. Y. Kim H. J. (2013). Effect of conjugated linoleic acid, mu-calpain inhibitor, on pathogenesis of Alzheimer’s disease. *Biochim. Biophys. Acta* 1831 709–718. 10.1016/j.bbalip.2012.12.003 23246577

[B41] Leggett J. D. Aspley S. Beckett S. R. D’Antona A. M. Kendall D. A. Kendall D. A. (2004). Oleamide is a selective endogenous agonist of rat and human CB_1_ cannabinoid receptors. *Br. J. Pharmacol.* 141 253–262. 10.1038/sj.bjp.0705607 14707029 PMC1574194

[B42] Liao Z. J. Liang R. S. Shi S. S. Wang C. H. Yang W. Z. (2016). Effect of baicalin on hippocampal damage in kainic acid-induced epileptic mice. *Exp. Ther. Med.* 12 1405–1411. 10.3892/etm.2016.3461 27588062 PMC4998122

[B43] Lichtman A. H. Hawkins E. G. Griffin G. Cravatt B. F. (2002). Pharmacological activity of fatty acid amides is regulated, but not mediated, by fatty acid amide hydrolase in vivo. *J. Pharmacol. Exp. Ther.* 302 73–79. 10.1124/jpet.302.1.73 12065702

[B44] Lipton S. A. Rosenberg P. A. (1994). Excitatory amino acids as a final common pathway for neurologic disorders. *N. Engl. J. Med.* 330 613–622. 10.1056/NEJM199403033300907 7905600

[B45] Lopatniuk P. Witkowski J. M. (2011). Conventional calpains and programmed cell death. *Acta Biochim. Pol.* 58 287–296. 21887410

[B46] Lubisch W. Hofmann H. P. Treiber H. J. Moller A. (2000). Synthesis and biological evaluation of novel piperidine carboxamide derived calpain inhibitors. *Bioorg. Med. Chem. Lett.* 10 2187–2191. 10.1016/S0960-894X(00)00430-3 11012026

[B47] Ma M. Ferguson T. A. Schoch K. M. Li J. Qian Y. Shofer F. S. (2013). Calpains mediate axonal cytoskeleton disintegration during Wallerian degeneration. *Neurobiol. Dis.* 56 34–46. 10.1016/j.nbd.2013.03.009 23542511 PMC3721029

[B48] Marsicano G. Goodenough S. Monory K. Hermann H. Eder M. Cannich A. (2003). CB_1_ cannabinoid receptors and on-demand defense against excitotoxicity. *Science* 302 84–88. 10.1126/science.1088208 14526074

[B49] Martinez-Gonzalez D. Bonilla-Jaime H. Morales-Otal A. Henriksen S. J. Velazquez-Moctezuma J. Prospero-Garcia O. (2004). Oleamide and anandamide effects on food intake and sexual behavior of rats. *Neurosci. Lett.* 364 1–6. 10.1016/j.neulet.2004.03.080 15193744

[B50] Mattson M. P. (2007). Calcium and neurodegeneration. *Aging Cell* 6 337–350. 10.1111/j.1474-9726.2007.00275.x 17328689

[B51] McGeer E. G. Zhu S. G. (1990). Lamotrigine protects against kainate but not ibotenate lesions in rat striatum. *Neurosci. Lett.* 112 348–351. 10.1016/0304-3940(90)90229-3 2141673

[B52] Mechoulam R. Deutsch D. G. (2005). Toward an anandamide transporter. *Proc. Natl. Acad. Sci. U.S.A.* 102 17541–17542. 10.1073/pnas.0508644102 16314567 PMC1308911

[B53] Mechoulam R. Fride E. Hanu L. Sheskin T. Bisogno T. Di Marzo V. (1997). Anandamide may mediate sleep induction. *Nature* 389 25–26. 10.1038/37891 9288961

[B54] Mehta A. Prabhakar M. Kumar P. Deshmukh R. Sharma P. L. (2013). Excitotoxicity: bridge to various triggers in neurodegenerative disorders. *Eur. J. Pharmacol.* 698 6–18. 10.1016/j.ejphar.2012.10.032 23123057

[B55] Miyamoto R. Shimakawa S. Suzuki S. Ogihara T. Tamai H. (2008). Edaravone prevents kainic acid-induced neuronal death. *Brain Res.* 1209 85–91. 10.1016/j.brainres.2008.02.064 18406401

[B56] Monory K. Massa F. Egertova M. Eder M. Blaudzun H. Westenbroek R. (2006). The endocannabinoid system controls key epileptogenic circuits in the hippocampus. *Neuron* 51 455–466. 10.1016/j.neuron.2006.07.006 16908411 PMC1769341

[B57] Mueller G. P. Driscoll W. J. (2009). Biosynthesis of oleamide. *Vitam. Horm.* 81 55–78. 10.1016/S0083-6729(09)81003-0 19647108

[B58] Murillo-Rodriguez E. Giordano M. Cabeza R. Henriksen S. J. Mendez Diaz M. Navarro L. (2001). Oleamide modulates memory in rats. *Neurosci. Lett.* 313 61–64. 10.1016/S0304-3940(01)02256-X 11684340

[B59] Nadler J. V. (1981). Minireview. Kainic acid as a tool for the study of temporal lobe epilepsy. *Life Sci.* 29 2031–2042. 10.1016/0024-3205(81)90659-7 7031398

[B60] Naidoo V. Karanian D. A. Vadivel S. K. Locklear J. R. Wood J. T. Nasr M. (2012). Equipotent inhibition of fatty acid amide hydrolase and monoacylglycerol lipase - dual targets of the endocannabinoid system to protect against seizure pathology. *Neurotherapeutics* 9 801–813. 10.1007/s13311-011-0100-y 22270809 PMC3480564

[B61] Nash D. R. Kaplan S. M. Norman A. B. Sanberg P. R. (1991). An evaluation of the possible protective effects of neonatal striatal transplants against kainic acid-induced lesions. *J. Neural Transplant. Plast.* 2 75–79. 10.1155/NP.1991.75 1831050 PMC2565087

[B62] Norenberg M. D. Rao K. V. (2007). The mitochondrial permeability transition in neurologic disease. *Neurochem. Int.* 50 983–997. 10.1016/j.neuint.2007.02.008 17397969 PMC4714712

[B63] Nozaki C. Markert A. Zimmer A. (2015). Inhibition of FAAH reduces nitroglycerin-induced migraine-like pain and trigeminal neuronal hyperactivity in mice. *Eur. Neuropsychopharmacol.* 25 1388–1396. 10.1016/j.euroneuro.2015.04.001 25910421

[B64] Patyar S. Medhi B. (2010). Lacosamide, a newer antiepileptic. *Neurosciences* 15 3–6. 20677583

[B65] Pisa M. Sanberg P. R. Corcoran M. E. Fibiger H. C. (1980). Spontaneously recurrent seizures after intracerebral injections of kainic acid in rat: a possible model of human temporal lobe epilepsy. *Brain Res.* 200 481–487. 10.1016/0006-8993(80)90938-5 7417826

[B66] Putkonen N. Kukkonen J. P. Mudo G. Putula J. Belluardo N. Lindholm D. (2011). Involvement of cyclin-dependent kinase-5 in the kainic acid-mediated degeneration of glutamatergic synapses in the rat hippocampus. *Eur. J. Neurosci.* 34 1212–1221. 10.1111/j.1460-9568.2011.07858.x 21978141

[B67] Quintanar L. Montiel T. Marquez M. Gonzalez A. Massieu L. (2012). Calpain activation is involved in acute manganese neurotoxicity in the rat striatum *in vivo*. *Exp. Neurol.* 233 182–192. 10.1016/j.expneurol.2011.09.032 21985864

[B68] Racine R. J. (1972). Modification of seizure activity by electrical stimulation. II. Motor seizure. *Electroencephalogr. Clin. Neurophysiol.* 32 281–294. 10.1016/0013-4694(72)90177-0 4110397

[B69] Reeta K. H. Mehla J. Pahuja M. Gupta Y. K. (2011). Pharmacokinetic and pharmacodynamic interactions of valproate, phenytoin, phenobarbitone and carbamazepine with curcumin in experimental models of epilepsy in rats. *Pharmacol. Biochem. Behav.* 99 399–407. 10.1016/j.pbb.2011.05.011 21641922

[B70] Rossini L. Garbelli R. Gnatkovsky V. Didato G. Villani F. Spreafico R. (2017). Seizure activity per se does not induce tissue damage markers in human neocortical focal epilepsy. *Ann. Neurol.* 82 331–341. 10.1002/ana.25005 28749594

[B71] Saatman K. E. Bozyczko-Coyne D. Marcy V. Siman R. McIntosh T. K. (1996). Prolonged calpain-mediated spectrin breakdown occurs regionally following experimental brain injury in the rat. *J. Neuropathol. Exp. Neurol.* 55 850–860. 10.1097/00005072-199607000-00010 8965100

[B72] Saussele T. (2008). Lacosamide. A new antiepileptic drug as adjunctive therapy in patients with partial-onset seizures. *Med. Monatsschr. Pharm.* 31 374–377. 18972867

[B73] Schmued L. C. Hopkins K. J. (2000). Fluoro-Jade B: a high affinity fluorescent marker for the localization of neuronal degeneration. *Brain Res.* 874 123–130. 10.1016/S0006-8993(00)02513-0 10960596

[B74] Shubina L. Aliev R. Kitchigina V. (2015). Attenuation of kainic acid-induced status epilepticus by inhibition of endocannabinoid transport and degradation in guinea pigs. *Epilepsy Res.* 111 33–44. 10.1016/j.eplepsyres.2015.01.003 25769371

[B75] Solomonia R. Nozadze M. Mikautadze E. Kuchiashvili N. Kiguradze T. Abkhazava D. (2008). Effect of oleamide on pentylenetetrazole-induced seizures in rats. *Bull. Exp. Biol. Med.* 145 225–227. 10.1007/s10517-008-0056-z 19023975

[B76] Sperk G. Lassmann H. Baran H. Seitelberger F. Hornykiewicz O. (1985). Kainic acid-induced seizures: dose-relationship of behavioural, neurochemical and histopathological changes. *Brain Res.* 338 289–295. 10.1016/0006-8993(85)90159-3 4027598

[B77] Verdon B. Zheng J. Nicholson R. A. Ganelli C. R. Lees G. (2000). Stereoselective modulatory actions of oleamide on GABA_A_ receptors and voltage-gated Na^+^ channels *in vitro*: a putative endogenous ligand for depressant drug sites in CNS. *Br. J. Pharmacol.* 129 283–290. 10.1038/sj.bjp.0703051 10694234 PMC1571835

[B78] Vilela L. R. Medeiros D. C. Rezende G. H. de Oliveira A. C. Moraes M. F. Moreira F. A. (2013). Effects of cannabinoids and endocannabinoid hydrolysis inhibition on pentylenetetrazole-induced seizure and electroencephalographic activity in rats. *Epilepsy Res.* 104 195–202. 10.1016/j.eplepsyres.2012.11.006 23352737

[B79] Vincent P. Mulle C. (2009). Kainate receptors in epilepsy and excitotoxicity. *Neuroscience* 158 309–323. 10.1016/j.neuroscience.2008.02.066 18400404

[B80] Vosler P. S. Brennan C. S. Chen J. (2008). Calpain-mediated signaling mechanisms in neuronal injury and neurodegeneration. *Mol. Neurobiol.* 38 78–100. 10.1007/s12035-008-8036-x 18686046 PMC2726710

[B81] Wang Q. Yu S. Simonyi A. Sun G. Y. Sun A. Y. (2005). Kainic acid-mediated excitotoxicity as a model for neurodegeneration. *Mol. Neurobiol.* 31 3–16. 10.1385/MN:31:1-3:003 15953808

[B82] Wang Y. Brittain J. M. Wilson S. M. Khanna R. (2010). Emerging roles of collapsin response mediator proteins (CRMPs) as regulators of voltage-gated calcium channels and synaptic transmission. *Commun. Integr. Biol.* 3 172–175. 10.4161/cib.3.2.10620 20585514 PMC2889978

[B83] Wang Y. Gu Z. L. Cao Y. Liang Z. Q. Han R. Bennett M. C. (2006). Lysosomal enzyme cathepsin B is involved in kainic acid-induced excitotoxicity in rat striatum. *Brain Res.* 1071 245–249. 10.1016/j.brainres.2005.10.074 16409994

[B84] Wang Y. Han R. Liang Z. Q. Wu J. C. Zhang X. D. Gu Z. L. (2008). An autophagic mechanism is involved in apoptotic death of rat striatal neurons induced by the non-N-methyl-D-aspartate receptor agonist kainic acid. *Autophagy* 4 214–226. 10.4161/auto.5369 18094625

[B85] Wu C. F. Li C. L. Song H. R. Zhang H. F. Yang J. Y. Wang Y. L. (2003). Selective effect of oleamide, an endogenous sleep-inducing lipid amide, on pentylenetetrazole-induced seizures in mice. *J. Pharm. Pharmacol.* 55 1159–1162. 10.1211/0022357021431 12956907

[B86] Wullner U. Standaert D. G. Testa C. M. Landwehrmeyer G. B. Catania M. V. Penney J. B. (1994). Glutamate receptor expression in rat striatum: effect of deafferentation. *Brain Res.* 647 209–219. 10.1016/0006-8993(94)91320-X 7922497

[B87] Yang J. Weimer R. M. Kallop D. Olsen O. Wu Z. Renier N. (2013). Regulation of axon degeneration after injury and in development by the endogenous calpain inhibitor calpastatin. *Neuron* 80 1175–1189. 10.1016/j.neuron.2013.08.034 24210906

[B88] Yoshimura T. Arimura N. Kaibuchi K. (2006). Molecular mechanisms of axon specification and neuronal disorders. *Ann. N. Y. Acad. Sci.* 1086 116–125. 10.1196/annals.1377.013 17185510

[B89] Zaczek R. Simonton S. Coyle J. T. (1980). Local and distant neuronal degeneration following intrastriatal injection of kainic acid. *J. Neuropathol. Exp. Neurol.* 39 245–264. 10.1097/00005072-198005000-00003 6154134

[B90] Zoppi S. Perez Nievas B. G. Madrigal J. L. Manzanares J. Leza J. C. Garcia-Bueno B. (2011). Regulatory role of cannabinoid receptor 1 in stress-induced excitotoxicity and neuroinflammation. *Neuropsychopharmacology* 36 805–818. 10.1038/npp.2010.214 21150911 PMC3055736

[B91] Zurmohle U. Herms J. Schlingensiepen R. Brysch W. Schlingensiepen K. H. (1996). Changes in the expression of synapsin I and II messenger RNA during postnatal rat brain development. *Exp. Brain Res.* 108 441–449. 10.1007/BF00227267 8801124

